# Gender considerations in One Health: a framework for researchers

**DOI:** 10.3389/fpubh.2024.1345273

**Published:** 2024-02-28

**Authors:** Alessandra Galiè, Anni McLeod, Zoë A. Campbell, Nicholas Ngwili, Zelalem G. Terfa, Lian F. Thomas

**Affiliations:** ^1^International Livestock Research Institute, Nairobi, Kenya; ^2^Independent Researcher, Edinburgh, United Kingdom; ^3^International Livestock Research Institute, Addis Ababa, Ethiopia; ^4^Institute of Infection, Veterinary and Ecological Sciences, University of Liverpool, Liverpool, United Kingdom; ^5^Royal (Dick) School of Veterinary Studies, University of Edinburgh, Edinburgh, United Kingdom

**Keywords:** gender, One Health, framework, *T. solium*, equity

## Abstract

One Health research and intervention outcomes are strongly influenced by gender dynamics. Women, men, girls, and boys can be negatively affected by gender-based disadvantage in any of the three One Health domains (animal, human, and environmental health), and where this occurs in more than one domain the result may be a compounding of inequity. Evidence worldwide shows that women and girls are more likely to suffer from such gender-based disadvantage. A thoughtfully implemented One Health intervention that prioritizes gender equity is more likely to be adopted, has fewer unintended negative consequences, and can support progress toward gender equality, however there is limited evidence and discussion to guide using a gender lens in One Health activities. We propose a framework to identify key gender considerations in One Health research for development – with a focus on Low-and Middle-Income Countries. The framework encourages developing two types of research questions at multiple stages of the research process: those with a bioscience entry-point and those with a gender entry-point. Gender considerations at each stage of research, institutional support required, and intervention approaches is described in the framework. We also give an applied example of the framework as it might be used in One Health research. Incorporation of gender questions in One Health research supports progress toward more equitable, sustainable, and effective One Health interventions. We hope that this framework will be implemented and optimized for use across many One Health challenge areas with the goal of mainstreaming gender into One Health research.

## 1 Introduction

There is currently a drive within the international community to integrate gender considerations into research, policy, and practice. This includes research and development activities in any of the three domains of One Health: animal, human, and environmental health. One Health recognizes the constantly evolving relationship between animals, humans, and the environment (1). The One Health High-Level Expert Panel (OHHLEP) states that “the health of humans, domestic and wild animals, plants, and the wider environment (including ecosystems) are closely linked and interdependent” (2). Motivation for integrating gender considerations is based on a growing appreciation that the social context in which problems occur influences research outcomes and therefore the effectiveness of development interventions in both disseminating relevant innovations and ensuring the benefits are equitably shared among the stakeholders.

The One Health Panel’s conceptualization of One Health (2) highlights the importance of equity and inclusivity, as do an earlier paper by (3) – although neither specifically mention gender- and a paper by Laing et al. (4). Van Patter et al. (5) argue that One Health researchers need to understand the political economies that often cause health disparities to progress toward equity. They further argue that integrating feminist thought into One Health research can help identify ‘the complexities and interconnections of power and difference that impact each of the three pillars of One Health’ (page 4).

Equitable access to appropriate innovations and to the associated benefits is paramount in gender equality, whereby men, women, boys, and girls have equal access to resources and opportunities that meet their needs, priorities, and interests. ‘Gender’ is a key organizing principle in society unconsciously used by people worldwide as a means of making sense of who we are in relation to the other (6). Gender, as well as other identity markers like age, ethnic group, religion, marital status, and caste influences the power you have in relation to others, the behaviors considered appropriate in a particular time or place, your roles and responsibilities, and your access to resources and opportunities (7, 8). Hierarchal relationships whereby some groups of people are given more privilege than others do not just affect interactions between individuals, they are entrenched in systems including human health and veterinary services, government institutions, schools and universities, and the economy. Gender analysis engages in such complex social dynamics which shape the differences in preferences, needs, and capacities we may see between women and men. Gender analysis is different from gender-disaggregated data collection which entails recording differences between women and men (the ‘what’) without any analysis of gender dynamics and norms that shape such gender differences (the ‘how’ and ‘why’ behind the ‘what’).

In this paper, we limit our discussion to women and men as the two main gender groups of interest based on the extensive body of literature on inter-gender differences between these two groups, particularly with respect to agriculture and rural livelihoods. However, we fully acknowledge that gender-diverse people often experience specific forms of discrimination (9). We believe that the process of developing questions on gender proposed in our framework would be valid for gender-diverse people and we would encourage other researchers to explore this possibility and include gender-diverse options.

In this paper, we provide some illustrative examples of the importance of gender considerations within key health challenges where a One Health approach is relevant. Women in lower- and middle-income countries, for example, are often disproportionately affected by zoonotic and infectious diseases due to their gender-based roles in domestic activities and animal production. In Uganda, practices associated with risk of transmission of Rift Valley fever such as handling raw meat or consumption of unpasteurized milk were influenced by gendered social roles (10, 11). In rural South Africa, men may be more exposed to risks associated with hunting and slaughtering wildlife and rodent control (12). In West Africa, outbreaks of Ebola, an emerging zoonotic disease, disproportionately affected the economic and social lives of young women, in particular. Closed schools led to increased pregnancies out of wedlock. After the 2015 outbreak, a policy established that ‘visibly pregnant girls’ would be unable to re-enroll in school, leading to a drop in girls’ access to education (13). Early evidence from the COVID-19 pandemic identified increased risk of mortality for men, possibly due to sex-based immunological differences or gender-based differences such as patterns of smoking or gendered hygiene practices (10).

An additional complexity is that due to social and gender norms (see definition below), health care workers in many countries are predominantly women, which put them at greater risk of contracting COVID-19 (14, 15). In rural Nepal, gender norms affected healthcare seeking behaviors and the likelihood of being prescribed and taking antibiotics, which are relevant to the (re)-emergence of antimicrobial resistance (16). In light of these and other studies, researchers have made a case for considering gender issues to minimizing food safety risks in livestock value chains (17) and for improved One Health research in pastoralist systems (18), noting the scarcity of frameworks that embed gender in One Health (19). While analytical frameworks for gender in human health already exist (20, 21), appropriate frameworks to support the integration of gender considerations across all three domains of One Health are currently lacking.

Building on the momentum from recent discussion and publications, we propose a framework highlighting key gender considerations across One Health research. This article is structured as follows:

- Section 2 defines One Health as applied to the development of the framework.
- Section 3 summarizes arguments for gender considerations in One Health research.
- Section 4 provides a brief description of methods.
- Section 5 describing an overview of the framework structure, gender considerations at each stage of one health research including institutional support required and intervention approaches.
- Section 6 presents an applied example of the framework as it might be used in One Health research on *Taenia solium*, the pork tapeworm.
- Section 7 provides the conclusion.

## 2 Defining One Health

**Figure 1:**
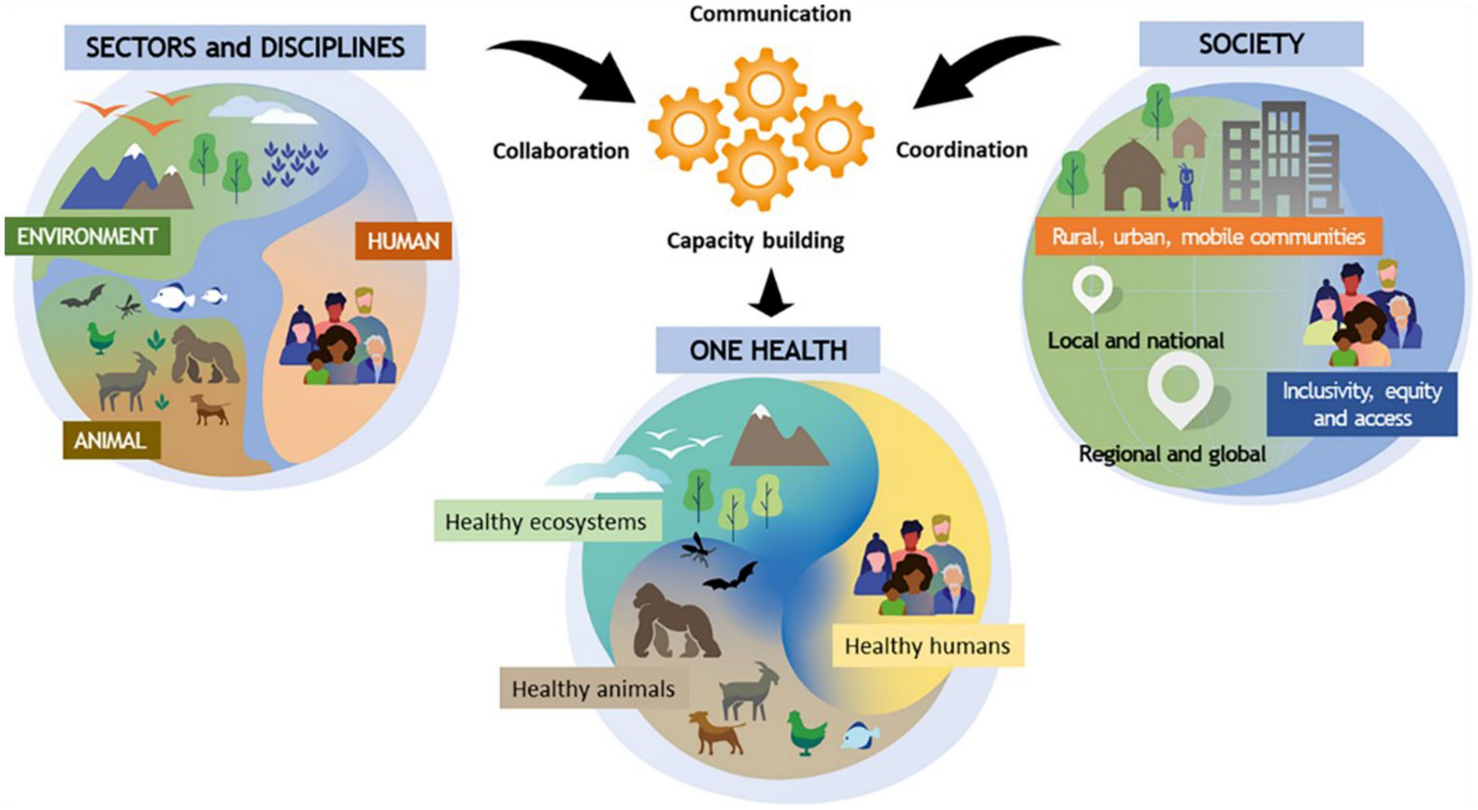
One Health as described by OHHLEP.

The definition of One Health has evolved over time; common ideas can be found across many sources, but emphasis has varied among institutions and according to context (22, 23). In 2021, a common definition was agreed by the One Health High Level Expert Panel (OHHLEP) (2). We used OHHLEP’s definition (below and Figure 1) as a starting point for developing a gender in One Health framework:

“One Health is an integrated, unifying approach that aims to sustainably balance and optimize the health of people, animals, and ecosystems.

It recognizes the health of humans, domestic and wild animals, plants, and the wider environment (including ecosystems) are closely linked and interdependent.

The approach mobilizes multiple sectors, disciplines, and communities at varying levels of society to work together to foster well-being and tackle threats to health and ecosystems, while addressing the collective need for clean water, energy and air, safe and nutritious food, taking action on climate change, and contributing to sustainable development.”

## 3 Gender considerations in One Health

A One Health approach “mobilizes multiple sectors, disciplines and communities at varying levels of society to work together to foster well-being and tackle threats to health and ecosystems” with ‘Equality, inclusivity and access’ being identified as a key enabler of this process (2). A recent compilation of One Health Core Competencies by the Network for Ecohealth and One Health included ‘Social, cultural and gender equity and inclusiveness’ as a core value for teams working in this space, re-emphasizing the need for explicit consideration of gender in One Health (4). A recent analysis demonstrated a correlation between improved gender equity indicators and positive indicators of social and ecosystem performance and, whilst not proving a casual link, demonstrates the complex interplay between social, health and environmental outcomes (24).

Embedding gender and other identity markers into One Health research aids in better understanding risks at the human-animal-environment interface (25) and identifying possible synergies, such as the potential to improve detection of emerging zoonotic diseases by widening access of previously underserved groups of livestock keepers to veterinary extension service providers. It also helps to identify possible compromises and trade-offs, which can aid in decision-making about which interventions to prioritize and maximize on the “added” value of One Health to inform policies toward addressing disproportionate burden of diseases.

One of the biggest challenges in including gender considerations in One Health research is the breadth and complexity of the One Heath subject area. As OHHLEP’s definition implies, a One Health approach can be applied to a multitude of research areas and topics. Each health domain includes gender-relevant issues, and additional issues arise when we consider the interfaces between domains of One Health. While gender considerations are relevant to each of the three health domains separately, in the framework proposed here we focus on gender considerations that are relevant at the interface between at least two or all the three health domains. Such considerations may reveal how gender-based disadvantage has a compounding effect across the three health domains. For example, disadvantaged access to and control over land for women or poorer individuals may expose them to environments with a higher risk of zoonotic disease and increase their chances of infection. If their livestock are infected by a zoonotic disease, these groups or individuals may lack access to animal health care, with negative consequences on the productivity of their livestock. Lost income from their livestock assets could influence their ability to seek medical care, exponentially increasing the negative impacts of such initial exposure to risky environments for these individuals – as compared to others who are better positioned to reduce exposure to risks by accessing needed resources and services.

Conversely, applying a gender lens while identifying and applying One Health solutions provides an opportunity to address gender-based discrimination across the three health domains and avoid the spiraling negative impacts described above. For example, improving vaccination coverage for zoonoses through a gender responsive approach to vaccine delivery and knowledge transfer has the potential to improve environmental and human health outcomes through improved animal health (26). Other types of inequality may influence the ways in which women and men, boys and girls, experience disadvantage or privilege (7). By taking into account identity markers (e.g., ethnicity, age, religion) that may be relevant in a given context, gender analysis can reveal axes and processes of inequality between women and men, and, also, across women and across men (21). An approach or solution that applies in one social context may work less well or be more difficult to apply in a different context. It is always advisable to conduct gender analyses with an intersectional lens, meaning that more than one source of inequality is considered. For a practical example of this approach (see Tavenner, 2022).

The complexity of One Health interventions means they inherently require trade-offs or compromises. For example, when controlling zoonotic diseases, there may be a range of possible options, such as vaccinating livestock or humans; treating livestock or humans; changing to livestock management practices; or applying hygiene measures in slaughterhouses, food retailers, or homes. Considering gendered impacts in addition to economic considerations can guide selection of control measures. A One Health intervention focusing on disease control and livestock intensification may increase household income and food security for men, but have no improvement in food security for children, with negative consequences on women’s workload and nearby wildlife communities. Including gender considerations gives a more complete picture of the potential benefits and consequences of the proposed activities, identifies how benefits and risk are distributed, and tries to suggest interventions that will allow everyone in the community to benefit.

Interventions or activities that change the status quo, redistribute resources, or require time or labor to implement may bring negative, unintended consequences for less privileged groups of people. On the other hand, interventions that reflect the status quo may reproduce and aggravate gender disadvantaged labor allocation and resource distribution patterns. In a meta-analysis of the impact of livestock interventions on indicators of women’s empowerment, the most common negative consequence was an increase in women’s workload (27), as reported by a dairy intensification project in Uganda, for example (28). Family members who do not see the benefits of an innovation may oppose its adoption in the household, undermining the effectiveness of an intervention, as in the case of East Coast Fever infection and treatment method in Kenya (29). Increase in workload can be an acceptable change if it brings commensurate benefits (30). Gender analysis can help appreciate what intervention is more likely to bring equitable benefits in a given context. At the bare minimum, a One Health intervention should aim to “do no harm,” which is only possible if a project is considering the impact of gender dynamics and other identity markers to monitor for and mitigate potential unintended consequences.

## 4 Methods

The framework was developed by the co-authors through a series of discussions, which allowed the drafting of an initial framework. From here, literature review and subsequent discussions were used to refine and finalize the final framework. The co-authors are all researchers with experience implementing gender and/or One Health research.

## 5 The gender in One Health framework

**Figure 2:**
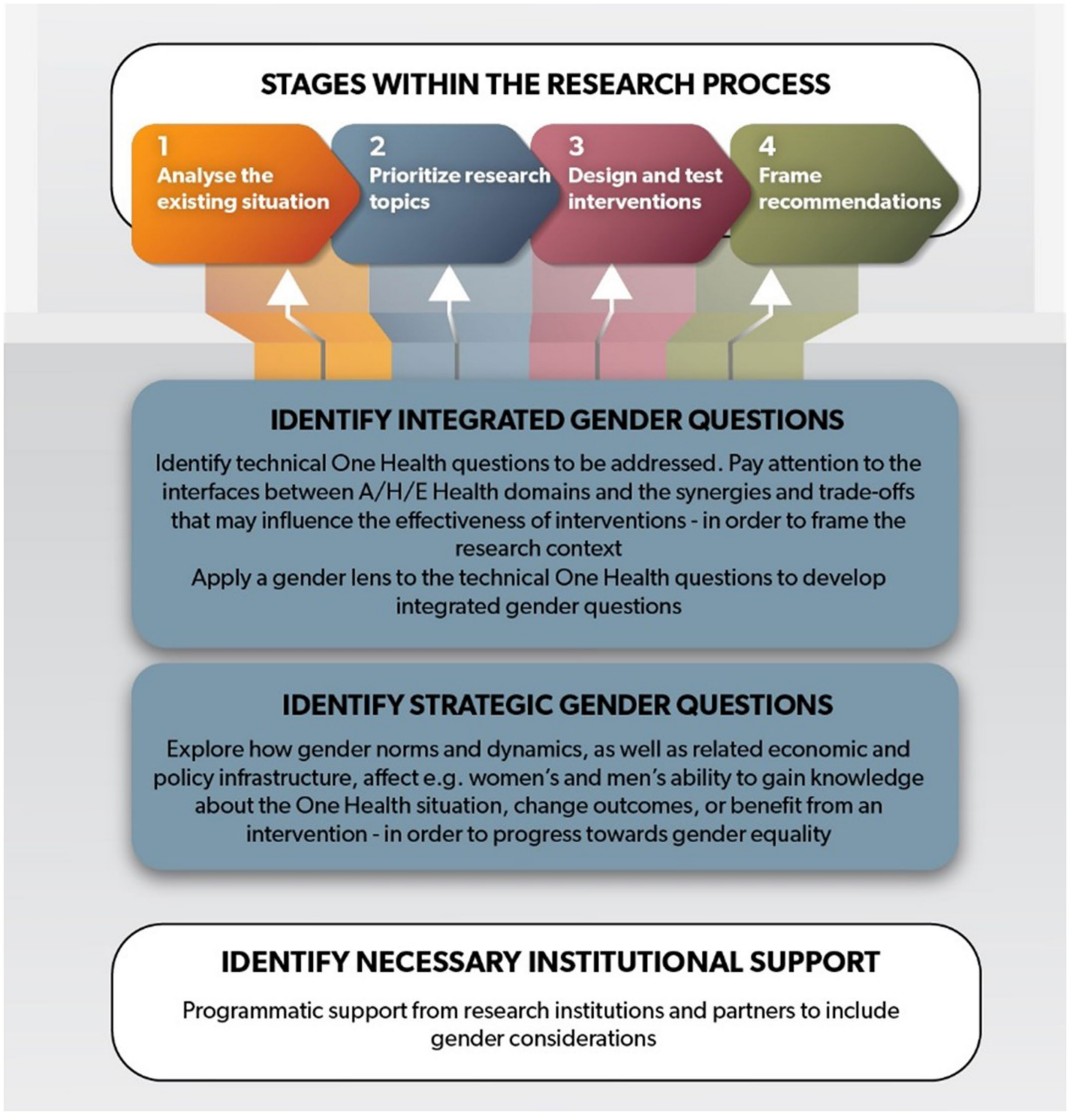
A framework for gender in One Health research. Every research stage involves identifying both integrated and strategic gender research questions and having underlying institutional support.

The framework is designed to support development of key gender questions for the application of a gender lens to One Health research and to frame a discussion among multi-disciplinary researchers. The questions we are proposing are as simple and non-prescriptive as we can make them, given the complexity of the topic. We also indicate programmatic measures to be put in place for gender research to be supported effectively. We do not provide models, tools, methods, suggestions for specific interventions, or policy recommendations because these can only be shaped by projects based on the evidence that emerges when applying the proposed research questions. We consider four stages of research for development, beginning from the diagnostic stage when (1) a given situation is explored with a gender lens in order to (2) identify priority issues that need to be researched; to (3) producing evidence that is necessary for designing One Health interventions that respond to gendered needs and priorities; and leading finally to (4) framing of recommendations for wider-scale changes (shown in the top of Figure 2).

Research projects that incorporate gender considerations early and throughout the lifecycle of the research tend to have more successful and meaningful gender analyses and have the opportunity to identify and mitigate negative unintended consequences or help progress toward gender equality (8). At each research stage, the framework encourages researchers to identify gender research questions in two categories. For the first category, a technical question from a One Health domain or the interface between two domains is the entry point to thinking about gender issues. Gender issues are integrated in the technical ones to improve the latter’s relevance and effectiveness. For example, “what are the animal and human health impacts of zoonotic disease x?” might be a technical question at the interface between human and animal health. Asking “how are these impacts apportioned between men and women, boys and girls?” adds gender nuance. These types of questions may feel familiar to bioscientists, veterinarians, and epidemiologists.

For the second category, gender is the entry point applied to a technical issue. In this case, the intended outcome is often progression toward gender equality (although such research can also be necessary to improve a technical intervention). For example, asking “to what extent do gender norms affect the ability of men, women, boys, and girls to reduce the impact they experience from zoonotic disease x?” address the same One Health issue as the previous questions, namely impact of disease, but it puts gender concerns at the heart of the question. These types of questions may feel more familiar to social scientists and gender researchers.

Both integrated and strategic research questions are explained below in more detail. As previously mentioned, formulating research questions is an activity best for an interdisciplinary team because of the breadth of experience required to identify research questions in both technical and social categories and the inherently interdisciplinary nature of the One Health approach. The extent to which the final set of research questions will span both categories depends on the goals and objectives of the project, the expertise of the implementors, and the financial support and timeline. Underlying the process is identifying appropriate institutional support (as shown in white in Figure 2). The specific research questions identified, and the extent of institutional support required will help determine the most appropriate intervention approach. We discuss each component of the framework in more detail below.

### 5.1 Gender considerations at each research stage

Next, we describe each of the four stages of research in more detail. They are presented as a linear flow, but in some cases, it may be a more iterative and circular process whereby the results in one stage suggest returning to a previous stage. Institutional support and intervention approaches are also discussed.

#### 5.1.1 Stage 1: analyze the existing situation

Here the existing situation is explored, to identify where research may be needed. This enables researchers and practitioners to understand differences in exposures, preferences and priorities of men, women, girls, boys taking into account other social identity markers. Integrating gender considerations at this stage ensures that technical One Health questions are positioned within their social context in later stages of the research. This is also a good time to begin anticipating potential consequences and trade-offs of any proposed interventions or activities.

#### 5.1.2 Stage 2: prioritize research topics

Integrating gender considerations at this stage affects the choice of One Health research topics by taking into account needs and preferences of men and women (across other individual markers). It also informs the way research is designed and undertaken and how interventions are implemented. For example, the choice of topic may be influenced by the priority that women, men, boys, and girls place on different livestock species, or their different exposure to health and environmental risks at home and along livestock value chains; other factors such as poverty, ethnicity and disability may also affect their priorities. The design of a research project on food safety or zoonotic disease control within households may be influenced by our understanding of gender norms, which affect household members’ knowledge of a problem and their ability to change what they do.

#### 5.1.3 Stage 3: design and test interventions

Assessing how a planned intervention may interact with local gender dynamics and norms, and consequently how effective and equitable it may be in the benefit it brings, is important to improve the intervention at the development stage. Assessing the performance of the actual intervention on the ground, with a gender lens, can help refine it for both effectiveness and equity outcomes for new rounds of ground testing before scaling. In the “Intervention approaches” section, we further describe two types of approaches that can be used in gender research.

#### 5.1.4 Stage 4: frame recommendations

Recommendations for the scaling of successful One Health interventions needs to be based on the evidence that emerges from the testing of interventions (stage 3). Because gender dynamics and norms are context specific, scaling of pilot interventions to other geographical areas can bring new complications as new gender dynamics may be at play. Recommendations need to carefully consider to what extent the findings produced by this framework can be generalized and how, in line with best practices of qualitative research.

#### 5.1.5 Integrated and strategic gender research questions

Here, we describe the two categories of gender research questions that can be considered for inclusion in more detail, as shown in the blue boxes in Figure 2.

“Integrated” gender questions explore the way problems and proposed interventions may differently affect women, men, boys, and girls. They take a technical livestock topic as an entry point and bring a gender lens to that topic. For example, integrating gender considerations into a tool to assess which forage varieties are a good fit for a community allows the identification of varieties appropriate for a given farm where women’s labor is more prevalent than men’s. Using a One Health example, gender roles in hunting wild animals, cleaning carcasses, and managing livestock may affect the way zoonotic diseases are passed between wildlife and livestock and to different members of a household; a successful intervention will need to identify, target, and communicate with those most involved in all of these roles.

“Strategic” gender questions are questions where gender issues are the focus of the study and the entry point to solving a One Health problem. They take a specific gender issue as an entry point and explore the wider gender norms and dynamics that drive the reasons for gendered differences. For example, a strategic gender question might aim to understand how changes in empowerment may differ between women and men, girls and boys in livestock communities. The decisions each person is able to make can affect the way they use environmental resources such as water or roadside forage, and the extent to which they are able to change what they do to make more effective use of resources or reduce their workload. Strategic gender questions may help to identify ways to leverage One Health interventions to progress toward gender equality.

**Figure 3:**
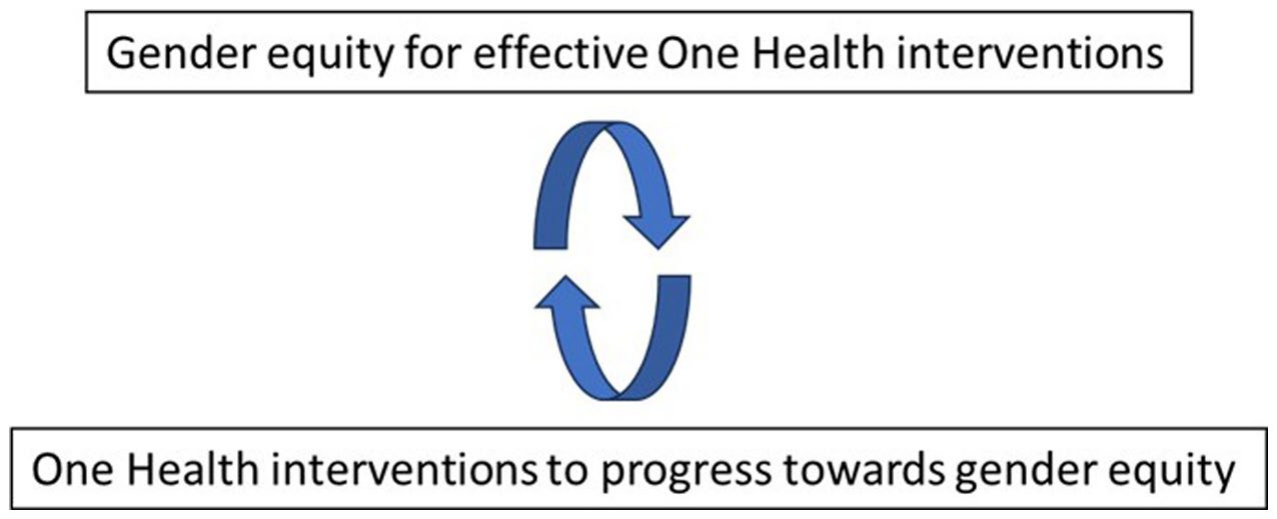
The interrelation between gender equity and One Health.

To appreciate the importance of both strategic and integrated gender questions it is important to acknowledge that gender equity and One Health interventions are mutually supporting goals. Equitable One Health interventions are more likely to be successful when all actors are supported to adopt them, and when they see the benefits they may gain. One Health interventions, on the other hand, are essential to progress toward gender equity to avoid that the possible spiraling effects of gender-based discrimination across the three health domains strongly disadvantage a gender group (Figure 3).

‘Gender norms’ are an important focus of gender strategic research. Gender norms are the unwritten rules that define and normalize as appropriate given identities, roles and actions for a gender. Gender norms affect who can do what kind of work, control what types of assets and make what level of decisions. They vary by context and time (8). Studying gender norms is essential to understand the processes behind gender-based discrimination.

Table 1 develops the concept of integrated and strategic gender questions by providing examples of typical questions that might be applied at each research stage of One Health.

**Table 1:** Key integrated and strategic gender questions at each research stage of One Health.

**Research stage**	**Integrated gender questions**	**Strategic gender questions**
1. Analyze the existing situation	How does the way men and women, girls and boys utilize the environment influence the transmission of disease x between wildlife, livestock, and humans? How do other identity markers like age, religion, or ethnic group influence environmental use and disease transmission? How do intra-household roles affect the way men and women, girls and boys are exposed to risks from foods of animal origin?	To what extent do gender norms affect the ways women, men, boys and girls interact with the environment thereby influencing the transmission of a given disease between wildlife, livestock and humans? To what extent do gender norms (across other relevant identity markers) affect the ability of men, women, boys, and girls to assess risk and act accordingly?
2. Prioritize research topics	Which impacts (human health, animal health, environmental health) are prioritized by women and by men (across other relevant identity markers)? What solutions/interventions would each group prefer?	How can the chosen interventions support the empowerment of women and girls (across identity markers) and enhance their capability to reduce both transmission of disease and the related impacts?
3. Design and test interventions	Are the identified interventions effective at reducing food safety risk for girls, boys, men and women within existing gender norms and customs?	To what extent do local gender norms and customs, and existing government policies, affect the way the chosen intervention impacts are distributed within households and communities?
4. Frame recommendations	How can we ensure that the recommended interventions are implemented and scaled with positive societal outcomes enjoyed by women, men, girls, boys (and across other relevant identity markers)?	

Many of the questions included in the framework and Table 1 are qualitative given the exploratory nature of gender research in One Health at this point in time (when little is known still, about the ways in which gender dynamics and norms are relevant to One Health). However, depending on the methods used, they could also be answered quantitatively. Qualitative evidence can support the identification of key gender issues that can then be further explored using a combination of qualitative and quantitative research methods (31).

#### 5.1.6 Institutional support

Institutional support is essential for integrating gender into research and effectively implementing the framework, so we explicitly include it as one of the components of the framework. It underpins all research stages. Institutional support includes arrangements with donors, capacity and willingness of the research institution to support gender research and interdisciplinary projects, and relationships with implementing partners which could include universities, non-governmental organizations (NGOs), government agencies, or private sector. An essential element of institutional support is ensuring that social science and gender expertise is included within research teams at all stages of the process. Following best practices in gender research has budget implications. For example, field research is designed in a way that allows men and women to be separately interviewed or separately consulted. This may involve additional staffing and inclusion of both men and women on data collection teams. Communities may be facilitated to discuss gender roles [such as the community conversations used by the One Health for Humans, Animals, Environment, and Livelihoods (HEAL) project (31)]. Analysis of qualitative data may require transcription and/or translation, which is time consuming and costly.

#### 5.1.7 Intervention approaches

In our framework, Stage 3 is about identifying key gender questions for the designing and testing interventions that respond to gendered needs, priorities, and dynamics. Here we provide additional information about the type of interventions that could be considered for a project after reviewing the integrated and strategic research findings.

Two main types of intervention approaches that respond to gendered evidence are accommodative and transformative. Accommodative approaches develop interventions that reflect the existing gender dynamics and norms (e.g., engaging farm women in identifying sick animals and men vets in providing and administering veterinary drugs in a community where this is the typical division of labor). Transformative approaches address and challenge gender discriminating norms, practices, and beliefs [e.g., involving women farmers in identifying sick animals and also striving to engage women – who may be interested in providing animal health services but may be discouraged by gender norms to do so – thereby providing business opportunities for them (32)]. In a project aimed at improving the uptake of animal vaccines in northern Ghana, addressing restrictive gender norms at the project outset was essential to create a conducive social environment for animal vaccines to be adopted by the communities (32). Restrictive norms discouraged women farmers from rearing livestock, assigned only men the burden of providing for the household, and prevented women from working as veterinarians. Gender strategic questions are particularly important to develop transformative approaches by exploring, for example, gendered aspirations and challenges to empowerment.

The next section shows how the framework can be applied in practice, using a worked example.

## 6 Applying the framework: a case study in zoonotic disease control

**Figure 4:**
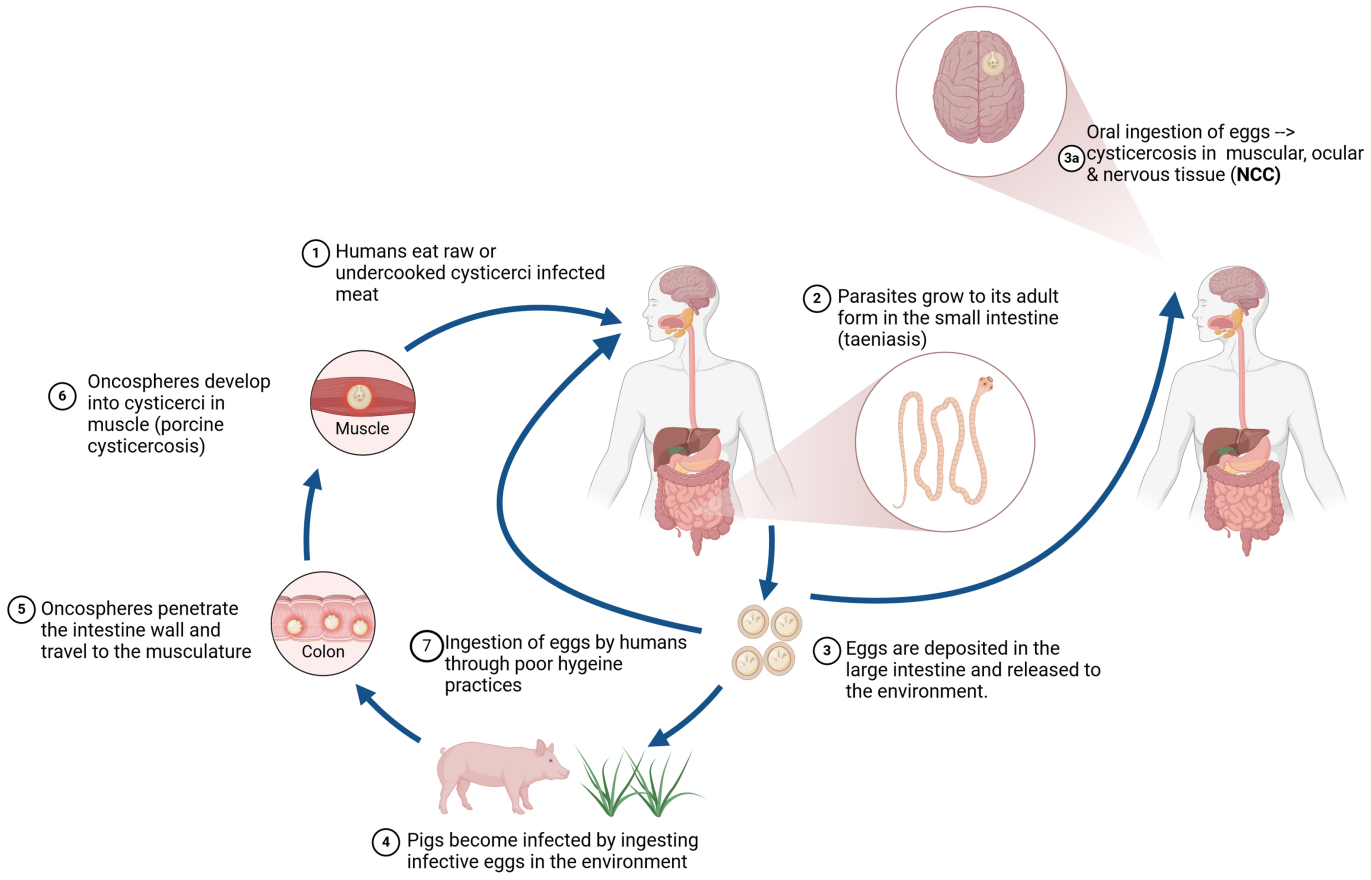
*Taenia solium* transmission routes. Reused with permission from (38).

To illustrate the application of the framework, we have used an example of research into the control of *Taenia solium* cysticercosis (*T. solium*), popularly known as the pork tapeworm. *T. solium* is an internal parasite which causes disease in humans and pigs. It is one of the leading causes of acquired epilepsy for people in endemic areas (33), primarily low- and middle-income countries in sub-Saharan Africa, Latin America, and Southeast Asia (34). The burden of this disease has been estimated as approximately 2.78 million Disability Adjusted Life Years (DALYs) globally (35). *T. solium* causes three diseases: taeniasis and neurocysticercosis in humans and porcine cysticercosis in pigs (36). Humans acquire taeniasis (a tapeworm infection) when they eat raw or undercooked pork meat contaminated with cysticerci, the larval form of *T. solium.* When ingested, the cysticerci establish in the intestine of humans, become adult tapeworms, and shed eggs in human feces that can infect in turn other humans and pigs by direct contact or by indirect contamination of water or food. Figure 4 illustrates typical transmission routes.

### 6.1 Research stage 1: analyze the existing situation

The likelihood of transmission of *T. solium* is influenced by factors including the proximity of people to the pigs they are rearing, how the pigs are kept (e.g., production system and housing), access to restrooms and sanitation facilities, hygiene practices, and dietary practices and preferences. Gender dynamics and the social context shape the differentiated roles women and men have in pig production with implications on exposure to disease. For example, in a recent study in Vietnam, both women and men were found to participate in the pig value chain but performed different tasks. Women did more routine husbandry activities such as cleaning pens and pork processing while men were more responsible for disease management, slaughtering, and large-scale farming (37).

Although gender disaggregated data on disease burden are not yet readily available, preliminary data suggest the burden of *T. solium* infections may vary by gender. In a study of hospital patients in Ecuador, there were differences in the presentation of neurocysticercosis, with female patients harboring more transitional cysts (those with inflammation surrounding them, indicative of a greater immune response). These transitional cysts put women at higher risk of developing servere complications such as encephalitis (39). It is not known the extent to which these differences are related to biological differences between males and females; to gendered differences in risk factors for infection such as access to healthcare, and other social determinants of health; or to the interaction of all of these factors.

When analyzing the existing situation, integrated and strategic gender questions can be used to explore some of the above issues, as demonstrated in Table 2.

**Table 2:** Applying the gender and one health framework to *Taenia solium*: Research Stage 1.

**1/Analyze the existing situation**
**One Health questions** **Interface: Animal health (AH)/Human health (HH)/Environmental health (EH)** What is the current societal burden of cysticercosis with respect to: human health and well-being economic burden animal health and welfare environmental impact What are the sources of risk for humans and animals?
**Integrated gender questions** Gendered questions about burden of disease: How is the human health burden of disease distributed across men, women, boys, and girls? Why? How are the economic burdens of disease shared among men, women, boys, and girls? Why? How is the animal burden of disease distributed across men and women livestock keepers? Why? (e.g., How does disease influence the productivity of livestock and access to livestock products men and women may differentially control?) How are the potential environmental impacts of cysticercosis control (e.g., ecotoxicity from anthelmintics) differently experienced by men and women? Why? How are health and economic burdens of disease affected by other identity markers? Gendered questions about sources of risk: What knowledge do women, men, and children have about risks from *Taenia solium* and ways to manage risks? Where do households invest in term of preventive actions? Do/would women and men invest differently? How? Why? Are there different investments in livestock owned or managed by women versus men? Within households and communities, who has access to preventive measures, e.g., using latrines, human anthelmintics? Why? Does infection of pigs lead to economic or food security risks (e.g., if affected pigs have a lower sale value)? Does this affect men and women differently?
**Strategic gender questions** If there are gendered differences in exposure to disease, managing risk, investment decision, and/ or access to preventive measures, what social norms contribute to these differences? How do gender roles in pork production and other parts of the pork value chain affect exposure to disease and ways of managing risk? Who in the household has the capability to make decisions that could affect risks and the burden of disease? (seek knowledge, make decisions about changing farming practices, change norms around hygiene and use of latrines, make decisions about where to invest, change norms around whose health is prioritized) To what extent do national policies influence gendered access to information (e.g., extension, schools) and human health preventive measures?
**Institutional support needed:** **Programmatic support** Research program planning that includes gender outcomes and targets. Research team management that facilitates interdisciplinary work. Dedicated budget for gender expertise within the research team for planning, field activities and data analysis. Dedicated budgets to operationalize gender-focused research activities. Gender considerations in logistics for fieldwork: what locations, timing, group composition, compensation, and communication will facilitate the engagement of women and men respondents? Planning of fieldwork to include both integrated and strategic gender questions. Gender capacity building and the appropriate gender balance in local field teams. **Wider institutional support** Recognition by all research partners of the importance of gender considerations in research. Community support for research activities that explore gender roles and norms.

### 6.2 Research stage 2: prioritize research topics

Stage 2 of the research process involves continuing to define and prioritize research topics, which in this case study, was interpreted as prioritizing control measures. Reducing the risk associated with pork consumption in the developing world is a public health priority as laid out in the World Health Organization roadmaps for Neglected Tropical Diseases (40, 41). Prevention and control measures to break the cycle of infection can potentially include household hygiene (e.g., use of latrines, handwashing), pharmaceutical treatment of infected pigs and/or humans, or vaccination of pigs (42). In recent years, research efforts have focused on testing known interventions and understanding the barriers to control. Appropriately identifying the people and groups to target is important to underpin success of interventions. The best way to do this is to consider the needs and preferences of the target audience even when selecting the choice of interventions to be tested. Table 3 shows examples of integrated and strategic gender questions that may guide the choice of *T. solium* control measures to be investigated. The necessary institutional support is also listed; although this may be similar at each stage, explicitly considering it at each stage ensures that it is not forgotten.

**Table 3:** Applying the gender and one health framework to *Taenia solium*: Research Stage 2.

**2/Prioritize research topics**
**One Health question** **Interface: AH/HH/EH** Which of the possible control strategies is likely to deliver the greatest value to society and the planet, and should therefore be prioritized? (e.g., latrines, treatment of animals, treatment of people, vaccination?)
**Integrated gender questions** Which human health impacts from cysticercosis are prioritized by women and by men? What type of solutions/interventions would they each prefer? Are there any anticipated negative consequences associated with the control strategy? If so, who would be most affected, how and why?
**Strategic gender questions** What type of intervention is most likely to support the empowerment of women & girls (across identity markers) & enhance their capability to reduce risks & impacts?
**Institutional support needed:** **Programmatic support** See Table 2. **Wider institutional support** Recognition by all research partners of the importance of gender considerations in research Community support for surveys to explore gender roles and norms.

### 6.3 Research stage 3: design and test interventions

In Stage 3, questions are asked so that interventions are designed and tested in gender-equitable ways. Based on the produced evidence projects can decide whether to take a gender accommodative and/or a gender transformative approach. For example, if the evidence shows that gender norms hinder men’s ability to engage in household hygiene practices, then an intervention may be designed to address such gender norms.

Site-specific contextual issues affect implementation and uptake of the interventions. Some aspects of the context include understanding the socioeconomic aspects encompassing gender related issues of the target population as described in Ngwili et al. (36). Gender norms and customs may also create barriers to adoption of control practices like the use of toilets and access to information on improved pig husbandry (36, 43). Addressing these gender-related issues requires a multifaceted approach. Gender considerations become very important due to issues related to decision making and control of resources within the households. For example, interventions focusing on health education may need to consider gender dynamics in the household (who makes decisions about health in the household) so that knowledge uptake translates to change in practices. Men may have control over who attends the training sessions even if they are not involved in actual implementation of the new practices. Some interventions such as the mass distribution of anthelminthic drugs often target children but will need support from parents (both men and women) to be effective (44, 45). These and other lessons on how failure to understand gender dynamics can affect implementation and uptake of interventions against *T. solium* have been discussed in two previous studies (25, 36).

Table 4 shows the framework applied to Stage 3 of the framework, designing and testing *T. solium* control measures.

**Table 4:** Applying the gender and one health framework to *Taenia solium*: Research Stage 3.

**3/Design and test interventions**
**One Health question** **Interface: AH/HH/EH** Which of the approaches to the prioritized interventions are most straightforward to implement? What is the societal and environmental impact (health, cost and sustainability) of each intervention?
**Integrated gender questions** Which interventions do men and women find easiest to implement and what characteristics of an intervention do they identify as important? What delivery mechanisms for the interventions are preferred by women and men? Why? Which interventions deliver the greatest benefit to women and to men (e.g., their own improved health; improved health of the pigs they manage)? Do men and women experience different costs or barrier to applying the interventions (e.g., costs of constructing a latrine, increased workload from cleaning the latrine)?
**Strategic gender questions** Whose capabilities need supporting and how, for the chosen interventions to benefit women and men across other social markers (e.g., ethnicity, age, religion etc.)? What actors in the system (from households to communities all the way to national policy makers) need to be involved to ensure that the intervention benefits women and men?
**Institutional support needed:** **Programmatic support** See Table 2. **Wider institutional support** Recognition by all research partners of the importance of gender considerations including, at a minimum, taking account of the availability of men and women for training sessions. Community support for gender-sensitive design of interventions including, for example, making it possible for women as well as men access new knowledge. National policies influencing gendered access to, e.g., veterinary services, e.g., distribution of pig vaccines, meat inspections or health services (e.g., anthelmintics for children).

### 6.4 Research stage 4: frame recommendations

Stage 4, the final research stage included in the framework, involves including considerations that help framing the recommendations arising from the previous three stages – these might involve, for example, scaling up of applied research to test implementations in a wider range of situation, or taking research findings into a delivery phase. Table 5 illustrates how research-to-delivery recommendations for *T. solium* control can acknowledge gender dynamics. At this stage, instead of integrated and strategic questions, we recommend including integrated and strategic gender considerations, which should influence the way the research findings are taken forward.

**Table 5:** Applying the gender and one health framework to *Taenia solium*: Research Stage 4.

**4/Frame recommendations**
**One Health recommendation** **Interface: AH/HH/EH** Technical recommendations (e.g., appropriate confining of pigs; appropriate anthelmintic use; vaccinate (where available); latrine provision; Water and Sanitation Hygiene (WASH) education on hygiene and cooking; site-appropriate anthelmintic use in humans) Policy recommendations (e.g., ensuring resources for meat inspection services; appropriate enforcement power for meat inspectors; make neurocysticercosis globally notifiable; consideration of cysticercosis endemicity within mass drug administration planning (e.g., mass drug administration in schools); policy environment for WASH).
**Integrated gender considerations** Recognize the most important gender considerations identified during research process that will affect who is likely to be involved in and benefit from scaling up and impact. Consider who may want to be involved and what measures are needed to ensure their involvement. Consider the gendered implications of any trade-offs, such as labor contributions for different interventions (e.g., young men for latrine construction, women for water collection to support WASH) vis-à-vis the acquired benefits. Ensure no one is harmed or worse off as a result of the intervention. Ensure that recommendations take into account gender considerations regarding access to facilities, resources or opportunities and benefits to avoid risk and identify equitable solutions.
**Strategic gender considerations** Ensure that the adopted interventions are implemented and scaled with positive societal outcomes enjoyed by women, men, girls, boys (across other relevant identity markers) Frame recommendations for targeted site-appropriate intervention taking into account gender norms that may affect the success and equitable outcomes of the intervention. Consider what systemic changes are needed for the intervention to be equitable (e.g., do labor policies need to be addressed? Is the engagement of community or religious leaders needed to achieve the desired goals?)
**Institutional support needed:** Support for all involved government service agents to promote appropriate gendered recommendations along the pork value chain e.g.: Community health and rural extension workers School administrators Inspectors at pork slaughterhouses Food safety inspectors at businesses serving pork

## 7 Conclusion

We have presented a framework to include gender considerations in One Health research. The framework helps to highlight both why gender considerations are relevant to One Health and how One Health can help progress toward gender equality; it suggests key gender questions that may be asked to appreciate how gender dynamics may interact with a One Health intervention; and it illustrates the application of the questions to research into the control of *T. solium*. Acknowledging, and gaining a deep understanding of the influence of gender dynamics can help interventions be adopted, minimize negative consequences, and support progression to a more equitable society. This framework supports conversations within interdisciplinary teams, emphasizing the need to consider gender throughout the lifecycle of the research project, develop both integrated and strategic gender questions, and to acquire appropriate institutional support. The inclusion of strategic research questions – which use gender as an entry-point and whose findings can inform gender transformative approaches – supports One Health teams to challenge the existing gender norms that limit the ability for some groups of people to adopt and benefit from interventions. Such efforts are vital if One Health is to improve ‘equality, inclusiveness and access’ as a societal mechanism for the improvement of health across human, animal and environmental domains as urged by the OHHLEP definition of One Health.

## Author contributions

AG: Conceptualization, Funding acquisition, Methodology, Project administration, Resources, Supervision, Visualization, Writing – original draft, Writing – review & editing. AM: Conceptualization, Methodology, Writing – original draft, Writing – review & editing. ZC: Visualization, Writing – review & editing. NN: Writing – review & editing. ZT: Writing – review & editing. LT: Conceptualization, Methodology, Resources, Supervision, Validation, Writing – original draft, Writing – review & editing.
